# LONP1 alleviates ageing‐related renal fibrosis by maintaining mitochondrial homeostasis

**DOI:** 10.1111/jcmm.70090

**Published:** 2024-09-11

**Authors:** Congxiao Zhang, Siman Shen, Li Xu, Man Li, Binyao Tian, Li Yao, Xinwang Zhu

**Affiliations:** ^1^ Blood Purification Center The Fourth People's Hospital of Shenyang, China Medical University Shenyang Liaoning P. R. China; ^2^ Department of Anesthesiology The Second Affiliated Hospital of Guangdong Medical University Zhanjiang Guangdong P. R. China; ^3^ Department of Laboratory Medicine The Second Affiliated Hospital of Guangdong Medical University Zhanjiang Guangdong P. R. China; ^4^ Department of Nephrology The First Affiliated Hospital of China Medical University Shenyang Liaoning P. R. China

**Keywords:** ageing, LONP1, m6A methyltransferase METTL3, mitochondrial homeostasis, renal fibrosis

## Abstract

Mitochondrial dysfunction is a pivotal event contributing to the development of ageing‐related kidney disorders. Lon protease 1 (LONP1) has been reported to be responsible for ageing‐related renal fibrosis; however, the underlying mechanism(s) of LONP1‐driven kidney ageing with respect to mitochondrial disturbances remains to be further explored. The level of LONP1 was tested in the kidneys of aged humans and mice. Renal fibrosis and mitochondrial quality control were confirmed in the kidneys of aged mice. Effects of LONP1 silencing or overexpression on renal fibrosis and mitochondrial quality control were explored. In addition, N6‐methyladenosine (m6A) modification and methyltransferase like 3 (METTL3) levels, the relationship between LONP1 and METTL3, and the impacts of METTL3 overexpression on mitochondrial functions were confirmed. Furthermore, the expression of insulin‐like growth factor 2 mRNA binding protein 2 (IGF2BP2) and the regulatory effects of IGF2BP2 on LONP1 were confirmed in vitro. LONP1 expression was reduced in the kidneys of aged humans and mice, accompanied by renal fibrosis and mitochondrial dysregulation. Overexpression of LONP1 alleviated renal fibrosis and maintained mitochondrial homeostasis, while silencing of LONP1 had the opposite effect. Impaired METTL3‐m6A signalling contributed at least in part to ageing‐induced LONP1 modification, reducing subsequent degradation in an IGF2BP2‐dependent manner. Moreover, METTL3 overexpression alleviated proximal tubule cell injury, preserved mitochondrial stability, inhibited LONP1 degradation, and protected mitochondrial functions. LONP1 mediates mitochondrial function in kidney ageing and that targeting LONP1 may be a potential therapeutic strategy for improving ageing‐related renal fibrosis.

## 1 INTRODUCTION

There is an increasing trend in the prevalence of chronic kidney disease (CKD).^1^ However, patients with CKD have a greater risk of death due to certain complications, such as electrolyte abnormalities, hypertension, anaemia, atherosclerosis and cardiac‐cerebrovascular disease.^2^ Ageing, as a particular inducible factor, has emerged among the numerous causes of CKD.^3^ The incidence of CKD in younger patients was 13% lower than that in older adults.^4^ Macrostructural changes, including increased surface roughness, decreased cortical volume and increased numbers and sizes of cysts, were observed in human kidneys at the age of 50–60 years.^5^ With advancing age, the kidney undergoes various impairments, such as a decrease in the glomerular filtration rate, a reduction in ion uptake and excretion capacity, and an increase in the ability to concentrate the urine, which gradually contributes to the progression of renal fibrosis. Therefore, gaining deeper insight into the mechanisms underlying ageing‐related renal fibrosis is crucial for the identification of appropriate therapies for CKD.

The kidney, a significant consumer of oxygen in the human body, relies heavily on ATP generated by mitochondrial oxidative phosphorylation to preserve normal functions. This organ is notably vulnerable to oxidative damage inflicted upon its mitochondria.^6^ Proximal tubule cells (PTCs) contain abundant mitochondria owing to their high energy demands.^7^, ^8^, ^9^ With ageing, mitochondrial dysregulation in aged PTCs has emerged as the principal cause of several kidney diseases.^10^, ^11^, ^12^ Tubular cells are central to kidney injury and repair, while senescent cells have a lower capacity for self‐repair and begin to produce matrix‐synthesizing molecules and proinflammatory cytokines, further accelerating renal fibrosis.^10^ Nevertheless, the molecular mechanisms underlying mitochondrial dysfunction in ageing‐related kidneys remain poorly understood.

Mitochondrial lon peptidase 1 (LONP1) is a well‐conserved soluble protease categorized among the ATPases related to various cellular activity families. LONP1 is responsible for preserving mitochondrial DNA (mtDNA) integrity and maintaining mitochondrial proteostasis through the selective breakdown of aberrantly and oxidatively damaged proteins, along with important rate‐limiting proteins.^13^ In addition, LONP1 is responsible for reprogramming mitochondrial metabolism and energetics under diverse cellular stressors, such as hypoxia, oxidative stress and nutrient deficiency.^14^, ^15^ Enhancing mitochondrial function via LONP1 regulation is likely crucial for several chronic and developmental conditions, including neurodegenerative diseases, heart ischemia and failure and malignancy.^15^ LONP1 protected against mitochondrial dysfunction and alleviated CKD by targeting 3‐hydroxy‐3‐methylglutaryl‐CoA synthase 2 (HMGCS2).^16^ There is limited understanding regarding the precise molecular mechanisms underlying dysfunctional LONP1 in aged‐induced kidney fibrosis.

In the present study, we initially posited that ageing‐related renal fibrosis is linked to decreased LONP1 levels. The levels of LONP1 and decreased mitochondrial function were observed in aged mouse kidneys with observably decreased ATP and mitochondrial generation and interstitial fibrosis. Further animal studies using adeno‐associated virus (AAV)‐Ksp‐LONP‐overexpressing accelerated aged mouse models revealed the regulatory role of LONP1 in age‐associated renal mitochondrial dysfunction and renal fibrosis. Reduced LONP1 was linked to decreases in m6A levels that regulate LONP1 degradation, which may provide a new mechanism and promising intervention target for delaying kidney ageing.

## 2 MATERIALS AND METHODS

### 2.1 Tissue samples

Twenty patients with renal cancer were enrolled at the First Hospital of China Medical University. Renal cortex tissues were carefully chosen from patients at least 5 cm away from the edge of the cancer tissues. None of the participants had received radiotherapy or chemotherapy before the surgery. The research procedure was approved by the Ethics Committee of the First Hospital of China Medical University, and all patients provided informed consent before the research. Subjects with obstructive nephropathy, renal insufficiency or other systemic diseases, such as diabetes and hypertension, were excluded.^17^ Individuals over 65 years of age are at high risk for developing end‐stage renal disease and drug‐related nephrotoxicity.^5^, ^18^ Therefore, all participants were categorized into two groups based on age: younger individuals (<65 years) and older individuals (≥65 years). The clinical characteristics of the participants are presented in Table S1.

### 2.2 Animal experimental design

Eight‐week‐, six and twenty‐four‐month‐old C57BL/6J mice were acquired from Charles River Laboratories (Beijing, China). For the mice ageing model, mice were euthanized at 6 months of age (representing the young group, *n* = 8) and at 24 months of age (representing the old group, *n* = 8).^10^ Eight‐week‐old mice received 500 mg/kg/day D‐gal subcutaneously for 2 months to establish an in vivo ageing model.^19^ An AAV‐packaged LONP1 or shLONP1 vector that targeted the specific promoter (cadherin) of renal tubular epithelial cells, referred to as AAV‐LNOP1 or AAV‐shLONP1, respectively, was obtained from GeneChem Company (Shanghai, China). AAV‐LNOP1 or AAV‐shLONP1 was inoculated into the renal pelvis of mice receiving D‐gal.

### 2.3 Cell culture, treatment and transfection

HK‐2 human renal tubule epithelial cells were cultured in DMEM/F12 (Gibco, USA) supplemented with fetal bovine serum (FBS) (10%, Gibco, USA) at 37°C under a humidified atmosphere with 5% CO_2_. D‐galactose (100 mM) was added to the cells for 72 h, and the resulting group was referred to as the D‐gal group. Lipofectamine 3000 (Invitrogen, Thermo Fisher Scientific) was utilized for cell transfection with the overexpression vector for LONP1 (SyngenTech, Beijing, China), methyltransferase‐like 3 (METTL3) (ChemicalBook), or insulin‐like growth factor 2 mRNA binding protein 2 (IGF2BP2) (SyngenTech). A scrambled sequence was used as a control.

### 2.4 Histological changes and immunohistochemistry (IHC)

The samples from humans or mice were sliced into 3 μm sections. Periodic acid Schiff (PAS), Sirius red and Masson's trichrome staining were performed to detect histological alterations in the well‐prepared samples. IHC was conducted in accordance with the manufacturer's directions. Thereafter, the primary antibodies and secondary antibodies were added to the samples.

### 2.5 Transmission electron microscopy

The samples were prepared as serial sections (80 nm) and subjected to transmission electron microscopy (TEM) to evaluate mitochondrial microstructure as previously described.^20^ The tissues were collected and fixed in phosphate buffer. Thereafter, the ultrathin sections were stained and detected under an electron microscope. The glomerular membrane thickness was examined with ImageJ 1.51 k software. The glomerular basement membrane (GBM) thickness was measured with the ImageJ plugin BoneJ software.

### 2.6 Western blot

Total protein was collected and quantified with a BCA protein assay kit (Roche Diagnostics, UK). Then, the samples were subjected to SDS–PAGE and transferred to nitrocellulose membranes (Pierce, Rockford, IL). Thereafter, the membranes were blocked, washed and incubated with primary antibodies against LONP1 (ab195352, Abcam), transcription factor A, mitochondria (TFAM) (NBP1‐71648, Novus), dynamin‐related protein 1 (Drp1) (ab184247, Abcam), IGF2BP2 (11601‐1‐AP, Proteintech), and α‐smooth muscle actin (α‐SMA) (ab5694, Abcam). Subsequently, secondary antibodies were used. β‐Actin was used as an internal control. The proteins were visualized with Millipore Immobilon Western Chemiluminescent HRP Substrate and quantified with ImageJ 1.42 software.

### 2.7 Mito‐tracker staining

After different stimulation conditions, HK‐2 cells or tissues were incubated with MitoTracker Red (200 nM, Thermo Fisher Scientific) at 37°C for half an hour. After the solution was removed, cell culture medium was added and a fluorescence microscope was used to obtain images.

### 2.8 Immunofluorescence (IF) staining

Frozen kidney sections were cut into 3 μm sections. The tissues and HK‐2 cells were fixed in paraformaldehyde and cold methanol/acetone, respectively. Following a 60‐min blocking step with 10% donkey serum (Thermo Fisher Scientific), the slides were subjected to immunostaining with primary antibodies at 4°C, including against LONP1 (66043‐1‐Ig, Proteintech), Drp1 (ab184247, Abcam), TFAM (NBP1‐71648, Novus) and cytochrome c oxidase (COX) IV (11802‐1‐AP, Proteintech) and then incubated with a secondary antibody. 4′,6‐Diamidino‐2‐phenylindole (DAPI) was used to stain the cell nuclei, and images were acquired with a microscope (LSM780, Leica Microsystems, Germany).

### 2.9 mtDNA level measurement

The levels of mtDNA were tested using RT–qPCR. Total DNA was prepared, and 10 ng of the DNA was used for qPCR. The mitochondrial ND1 gene (mtND1) and the mitochondrial gene COX2 were used to determine mtDNA copy numbers in humans and mice, respectively. Nuclear beta‐2 microglobulin (β2 M)^21^ and ribosomal protein s18 (RPS18)^22^ were used as internal controls.

### 2.10 ATP

The ATP content was quantified by a commercial ATP colorimetric assay kit (K354‐100, BioVision). Briefly, fresh tissue or cell suspension samples were lysed, centrifuged at 12,000 rpm at 4°C for 5 min and collected. The supernatant (20 μL) was mixed with the luciferase reagent (100 μL), and then the concentration of ATP was determined using a microplate luminometer (Promega, Madison, WI, USA).

### 2.11 Mitochondrial oxygen consumption rate

Mitochondrial oxygen consumption rate (OCR) was assessed with a Seahorse X96 Extracellular Flux Analyser. HK‐2 cells were plated in cell culture microplates with base medium and incubated. Thereafter, the medium was replaced, and the cells were treated with oligomycin (1 μM), FCCP (0.5 μM) or a combination of antimycin A (0.5 μM) and rotenone (0.5 μM). The parameters were examined, and the values are presented as the OCR (pmol/min/μg protein).

### 2.12 m6A modification quantification

The quantification of m6A modifications was conducted according to the manufacturer's guidelines. Briefly, total RNA was isolated, and 200 ng of total RNA was bound to 96‐well plates. After removing the binding solution, the plates were washed, the anti‐m6A antibody was added, and the plates were washed again. Thereafter, the developer solution was added and incubated in the dark, followed by the addition of the stop solution. The optical density (OD) was measured using a microplate reader.

### 2.13 RNA immunoprecipitation (RIP)‐PCR and sequencing analysis

A Magna RIPTM RNA‐Binding Protein Immunoprecipitation Kit (17–701, Millipore, Burlington, MA, USA) was used for RIP. Briefly, total RNA was prepared and incubated with oligo(dT)‐attached magnetic beads overnight coated with specific antibodies against METTL3 (10573‐1‐AP, Proteintech), IGF2BP2 (11601‐1‐AP, Proteintech), or rabbit IgG (IgG, Millipore). The immunoprecipitated RNA was extracted, and PCR was performed to determine the relative interactions. METTL3‐mediated mRNA was prepared from the D‐gal and D‐gal + METTL3 overexpression groups. Next‐generation sequencing was performed on a MiSeq sequencer (RiboBio, China).

### 2.14 Methylated RNA immunoprecipitation (MeRIP)‐qPCR

RNA was prepared and fragmented into ∼200 bp fragments using RNA fragmentation reagents (AM8740, Thermo Fisher Scientific). Approximately 1/10 of the fragmented RNA served as the input control. Protein A/G magnetic beads were mixed with an anti‐m6A antibody (ab151230, Abcam) in immunoprecipitation buffer. Anti‐IgG served as a negative control. Thereafter, the m6A‐containing RNAs were eluted, purified, and subjected to RT–qPCR analysis.

### 2.15 RNA stability assays

HK‐2 cells were incubated until 70%–80% confluency was reached. Thereafter, actinomycin D (HY‐17559, MedChemExpress) (5 μg/mL) was added to the plates to prevent the synthesis of intracellular RNA. At the indicated times, total RNA was isolated, and the mRNA levels of LONP1 were detected by RT–qPCR.

### 2.16 Statistical analysis

The resulting data are presented as the mean ± standard deviation (SD). The experiments were carried out with triplicate replicates. Two‐tailed unpaired Student's *t*‐tests were used for between‐group comparisons, and one‐way analysis of variance (ANOVA) followed by the Bonferroni correction was used for multiple comparisons. IBM SPSS Statistics 15.0 software was used for the data analysis. Significance was indicated by a *p* < 0.05.

## 3 RESULTS

### 3.1 LONP1 is decreased in aged human kidneys

**FIGURE 1:**
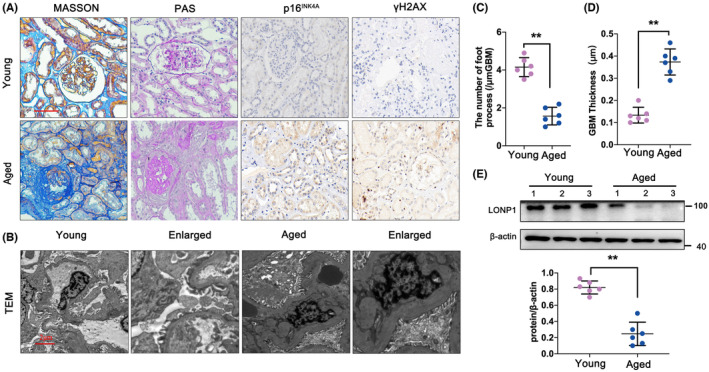
LONP1 is decreased in aged human kidneys. (A) PAS, Masson, and IHC staining for p16^INK4A^ and γH2AX in the aged and young groups. The scale bar represents 50 μm; (B–D). Representative TEM images of the aged and young groups. (E) Protein levels of LONP1 in the aged and young groups. **p < 0.01 versus the Young group by Student's *t*‐test. LONP1, lon protease 1; PAS, periodic acid–Schiff; IHC, immunohistochemistry; TEM, transmission electron microscopy.

To identify the important functions of LONP1 during kidney ageing, young and aged human kidney tissues were collected. PAS and Masson staining revealed nephrosclerosis, focal tubular atrophy, renal interstitial fibrosis and extracellular matrix (ECM) accumulation in the aged group (Figure 1A). The expression of P16^INK4A^ and γH2AX, two typical senescence‐related protein markers, was obviously greater in the aged group than in the young group (Figure 1A). In terms of renal ultrastructure, compared with that in the young group, the number of podocytes in the foot process was significantly decreased (*p* < 0.01), while the GBM thickness was significantly increased (*p* < 0.01) in the aged group (Figure 1B–D). Furthermore, the expression of LONP1 was examined in kidney tissue, and the results revealed that the expression of LONP1 was significantly lower in aged kidneys than in young kidneys (*p* < 0.01) (Figure 1E).

### 3.2 LONP1 is decreased in aged kidney mice, accompanied by mitochondrial dysfunction

**FIGURE 2:**
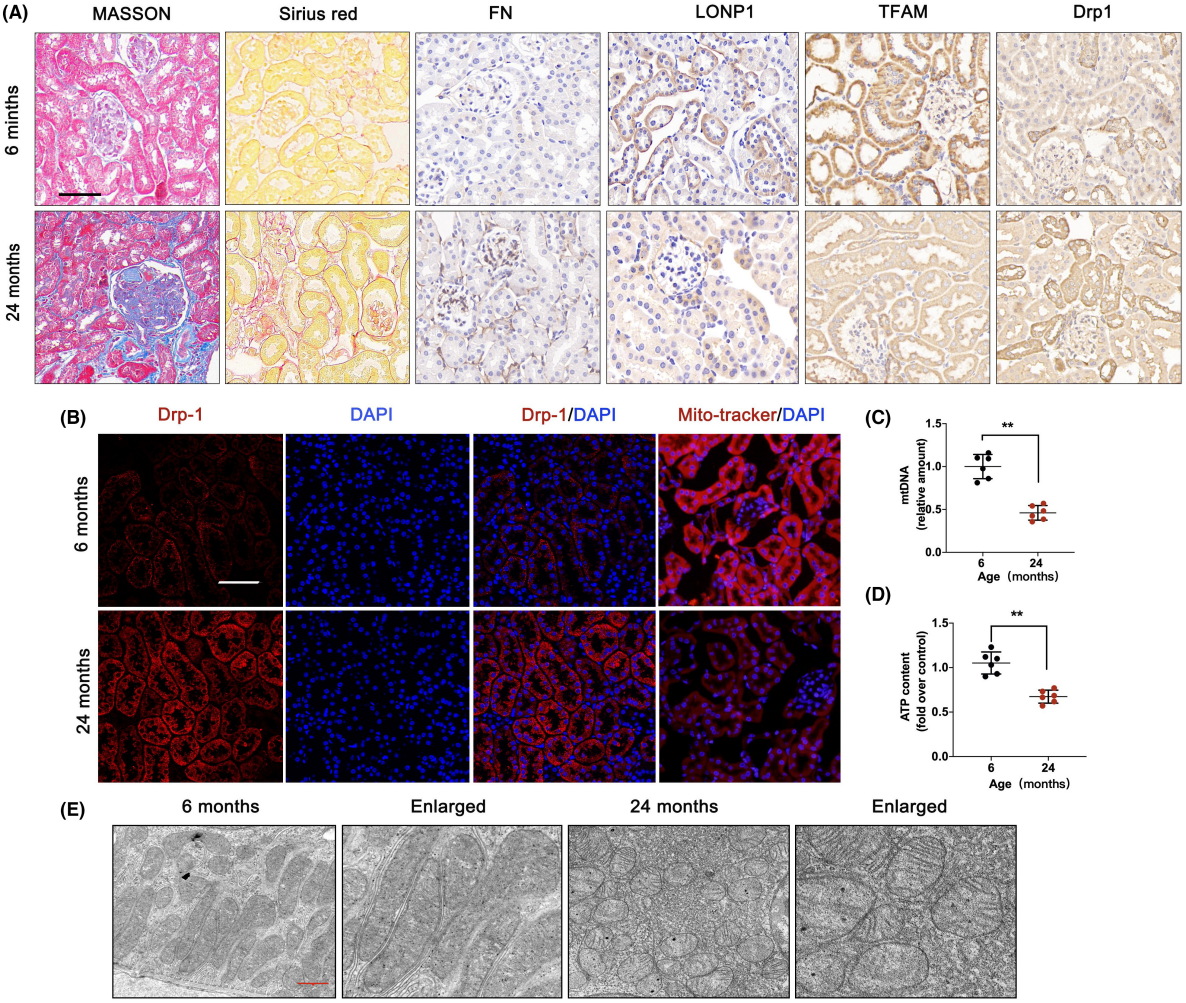
LONP1 is decreased in aged kidney mice, accompanied by mitochondrial dysfunction. (A) Masson, Sirius red and IHC staining for FN, LONP1, TFAM and Drp1 in the 6‐ and 24‐month groups. The scale bar is 50 μm; (B) double immunostaining of Drp‐1 with MitoTracker and counterstaining with DAPI were performed in the 6‐ and 24‐month groups. The scale bar represents 50 μm; (C, D) mtDNA levels and ATP content in the 6‐ and 24‐month groups; (E) mitochondrial ultrastructure in the 6‐ and 24‐month groups by TEM observation. **p < 0.01 versus the 6‐month group by Student's *t*‐test. LONP1, lon protease 1; IHC, immunohistochemistry; TFAM, transcription factor A, mitochondrial; Drp1, dynamin related protein 1; DAPI, 4′,6‐diamidino‐2‐phenylindole; IF, immunofluorescent; mtDNA, mitochondrial DNA; TEM, transmission electron microscopy.

The expression of LONP1 and mitochondrial quality control homeostasis were investigated in 6‐ and 24‐month‐old mice. Age‐associated ECM accumulation and fibrosis were confirmed. As shown in Figure 2A, the accumulation of ECM was obviously greater in the renal tubulointerstitial space and glomerular area of the aged kidney. IHC results showed that the expression of fibronectin (FN), an important fibrosis‐associated protein, was markedly increased in 24‐month‐old mice. In addition, the results revealed that the expression of LONP1 and TFAM was markedly reduced and that the expression of Drp1 was markedly augmented in the 24‐month group. The expression of Drp1 was further confirmed by IF analysis (Figure 2B). Furthermore, the intensity of the MitoTracker Red‐positive signal, the mtDNA level (*p* < 0.01) and the ATP content (*p* < 0.01) were significantly decreased in the 24‐month‐old group (Figure 2B–D). TEM analysis revealed a decreased mitochondrial count and damaged structure characterized by swelling and disorganized cristae in the 24‐month‐old group (Figure 2E). The results indicated that kidney ageing was accompanied by mitochondrial dysfunction and increased renal fibrosis with downregulated LONP1.

### 3.3 Silencing of LONP1 aggravates ageing‐related renal fibrosis and mitochondrial dysfunction in vivo

**FIGURE 3:**
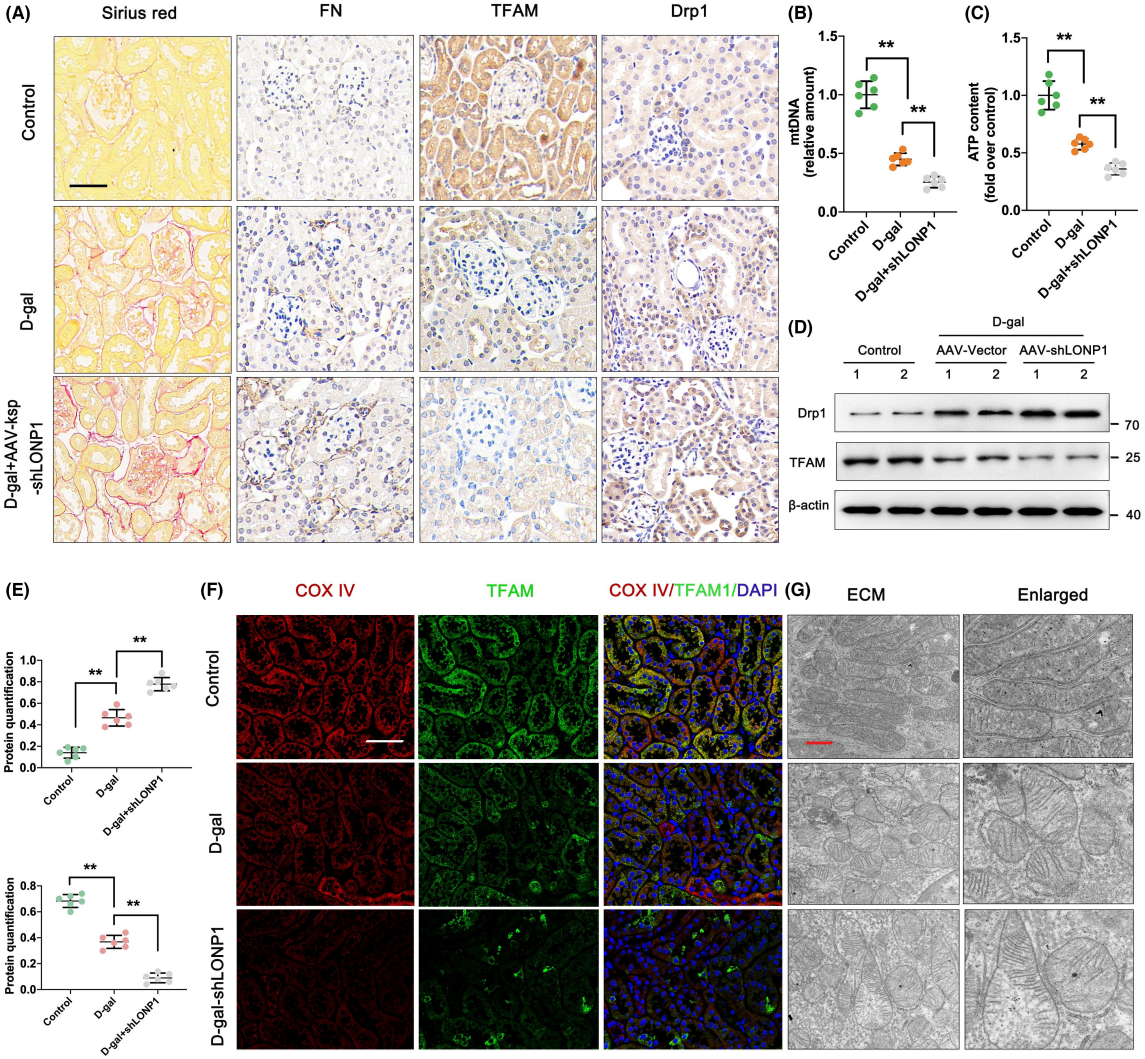
Silencing of LONP1 aggravates ageing‐related renal fibrosis and mitochondrial dysfunction in vivo. (A) Sirius red and IHC staining for FN, TFAM and Drp1 in the control, D‐gal and D‐gal + AAV‐shLONP1 groups. The scale bars are 50 μm; (B, C) mtDNA levels and ATP content in the control, D‐gal and D‐gal + AAV‐shLONP1 groups; (D, E) protein levels of TFAM and Drp1 in the control, D‐gal and D‐gal + AAV‐shLONP1 groups; (F) immunostaining for COX IV (red) and TFAM (green) and counterstaining with DAPI (blue) in the control, D‐gal and D‐gal + AAV‐shLONP1 groups. The scale bar represents 50 μm. (G) Mitochondrial ultrastructure in the control, D‐gal and D‐gal + AAV‐shLONP1 groups as determined by TEM. **p < 0.01 versus the D‐gal group by one‐way ANOVA. LONP1, lon protease 1; IHC, immunohistochemistry; FN, fibronectin; TFAM, transcription factor A, mitochondrial; Drp1, dynamin related protein 1; mtDNA, mitochondrial DNA; AAV, adeno‐associated virus; COX, cytochrome c oxidase; DAPI, 4′,6‐diamidino‐2‐phenylindole; IF, immunofluorescent; SD, standard deviation; ANOVA, analysis of variance.

To investigate the functional role of LONP1 in aged kidneys, D‐gal‐treated mice were injected with AAV‐shLONP1, and the degree of renal fibrosis and mitochondrial function were subsequently examined. According to the results shown in Figure 3A, IHC and Sirius red staining revealed that the accumulation of ECM and the expression of FN and Drp1 were distinctly amplified in the interstitial region of the kidney by treatment with D‐gal compared to those in the control group, whereas these changes were further aggravated by silencing of LONP1. The expression of FN showed opposite results. In addition, the mtDNA and ATP contents were significantly reduced by D‐gal treatment, and they were further reduced by LONP1 knockdown (*p* < 0.01) (Figure 3B,C). The expression of TFAM and Drp1 was further confirmed by Western blotting (Figure 3D,E). The colocalization of COX IV and TFAM translocases was examined. D‐gal‐mediated knockdown of LONP1 destroyed the mitochondrial network and prevented mitochondrial generation (Figure 3F). Additionally, mitochondrial swelling and cristae collapse were observed in the D‐gal group, whereas shLONP1 further accelerated mitochondrial dysfunction (Figure 3G). These data suggested that silencing LONP1 aggravated ageing‐related kidney fibrosis and mitochondrial dysfunction.

### 3.4 LONP1 overexpression ameliorates ageing‐related kidney fibrosis and mitochondrial dysfunction in vivo

**FIGURE 4:**
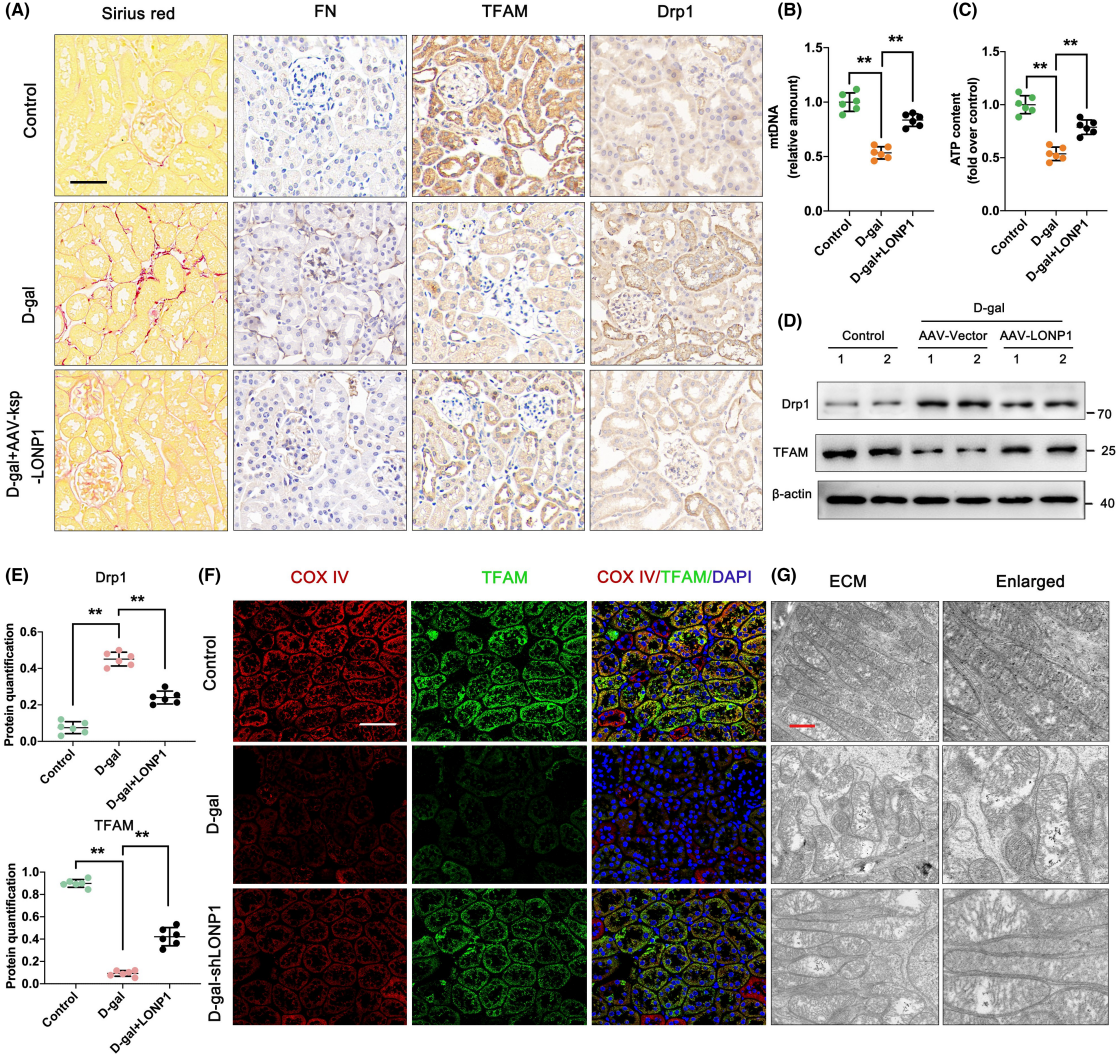
LONP1 overexpression ameliorates ageing‐related renal fibrosis and mitochondrial dysfunction in vivo. (A) Sirius red and IHC staining for FN, TFAM and Drp1 in the control, D‐gal and D‐gal + AAV‐LONP1 groups. The scale bars are 50 μm; (B, C) mtDNA and ATP contents in the control, D‐gal and D‐gal + AAV‐LONP1 groups; (D, E) protein levels of TFAM and Drp1 in the control, D‐gal and D‐gal + AAV‐LONP1 groups; (F) immunostaining for COX IV (red) and TFAM (green) and counterstaining with DAPI (blue) in the control, D‐gal and D‐gal + AAV‐LONP1 groups. The scale bar represents 50 μm. (G) Mitochondrial ultrastructure in the control, D‐gal and D‐gal + AAV‐LONP1 groups as determined by TEM. **p < 0.01 versus the D‐gal group by one‐way ANOVA. LONP1, lon protease 1; IHC, immunohistochemistry; FN, fibronectin; TFAM, transcription factor A, mitochondrial; Drp1, dynamin related protein 1; mtDNA, mitochondrial DNA; AAV, adeno‐associated virus; COX, cytochrome c oxidase; DAPI, 4′,6‐diamidino‐2‐phenylindole; IF, immunofluorescent; ANOVA, analysis of variance.

Furthermore, we overexpressed LONP1 in mice treated with D‐gal and then measured the degree of renal fibrosis and mitochondrial function. As shown in Figure 4A, the overexpression of LONP1 decreased ECM accumulation and attenuated renal interstitial fibrosis. Western blot and IHC assays revealed that LONP1 overexpression significantly decreased the expression of FN and Drp1 but increased the expression of TFAM (Figure 4A,D,E). In addition, the levels of the mitochondrial function‐related markers mtDNA and ATP were greatly increased in the AAV‐LONP1 group compared with the D‐gal group (Figure 4B,C). Compared with that in the D‐gal group, the fluorescence intensity in the AAV‐LONP1 group increased (Figure 4F). Regarding mitochondrial ultrastructure, overexpression of LONP1 reversed the D‐gal‐induced decreases in mitochondrial number and mitochondrial damage (Figure 4G). Our data showed that the overexpression of LONP1 mitigated ageing‐related renal fibrosis and mitochondrial dysfunction.

### 3.5 LONP1 attenuates D‐gal‐induced mitochondrial dysfunction in vitro

**FIGURE 5:**
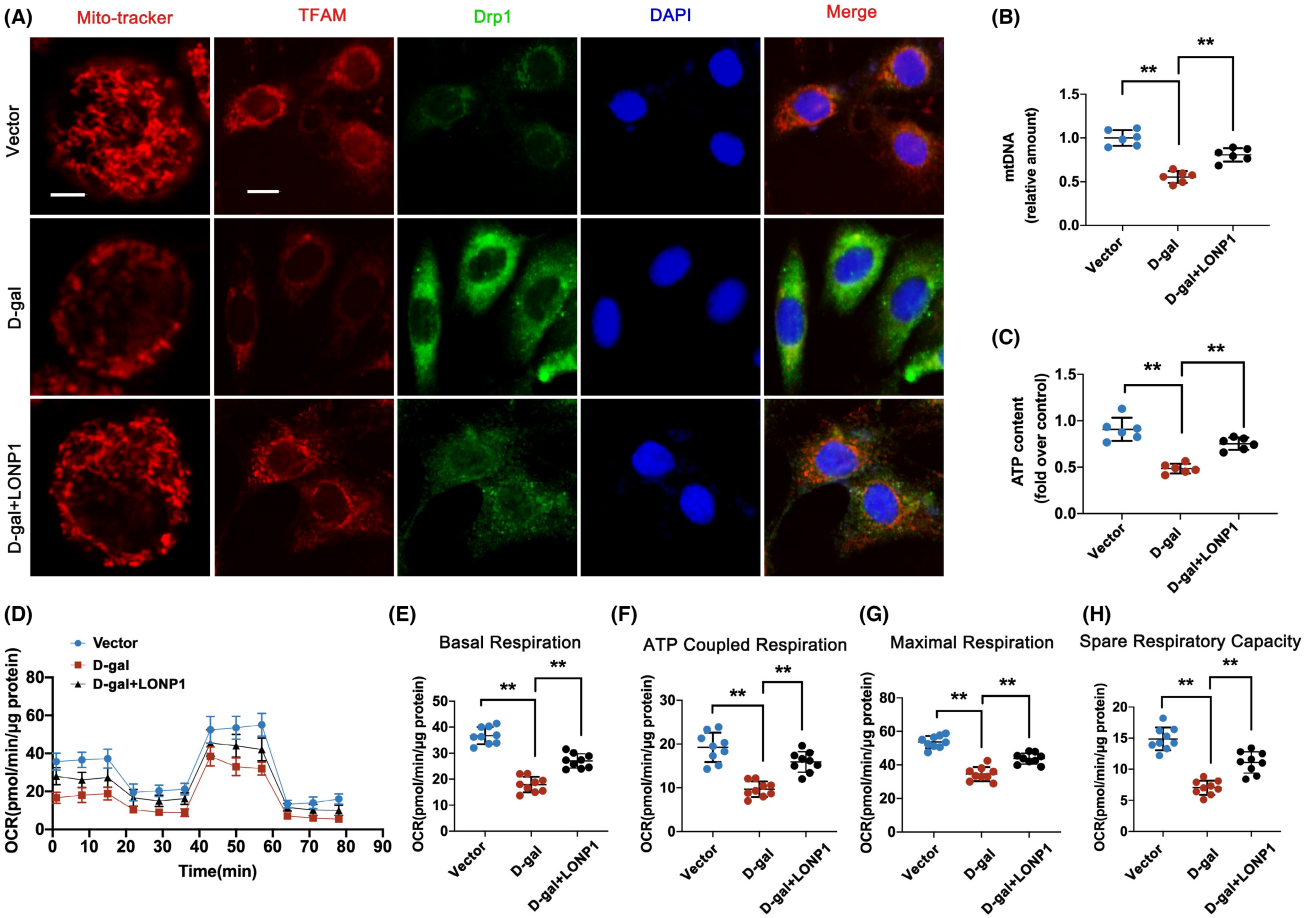
LONP1 attenuates D‐gal‐induced mitochondrial dysfunction in vitro. (A) Representative fluorescence micrographs obtained by MitoTracker deep red staining (scale bar = 10 μm) or double immunostaining of TFAM (red) and Drp1 (green), counterstained with DAPI (blue) (scale bar = 50 μm) in HK‐2 cells; (B, C) mtDNA levels and ATP content in the vector, D‐gal and D‐gal+LONP1 groups of HK‐2 cells; (D–H) the mitochondrial OCR in the vector, D‐gal and D‐gal+LONP1 groups of HK‐2 cells. **p < 0.01 versus the D‐gal group by one‐way ANOVA. LONP1, lon protease 1; TFAM, transcription factor A, mitochondrial; Drp1, dynamin related protein 1; DAPI, 4′,6‐diamidino‐2‐phenylindole; mtDNA, mitochondrial DNA; OCR, oxygen consumption rate; ANOVA, analysis of variance.

The effects of LONP1 on cellular mitochondrial function in HK‐2 cells were further examined. The transfection efficiency of LONP1 was shown in Figure S1. The mitochondrial morphology of the vector group showed a filamentous shape, which was transformed into short rods by D‐gal and reversed by overexpression of LONP1. In addition, LONP overexpression obviously increased the expression of TFAM but reduced the level of Drp‐1 (Figure 5A). Moreover, HK‐2 cells overexpressing LONP1 had greater mtDNA levels (*p* < 0.01) and ATP levels (*p* < 0.01) than did HK‐2 cells in the D‐gal group (Figure 5B,C), accompanied by increased basal respiration (*p* < 0.01), ATP‐coupled respiration (*p* < 0.01), maximal respiration (*p* < 0.01) and spare respiration capacity (*p* < 0.01) (Figure 5D–H). These data suggested that the overexpression of LONP1 alleviated mitochondrial dysfunction in vitro.

### 3.6 METTL3 regulates the aberrant m6A modification of LONP1

**FIGURE 6:**
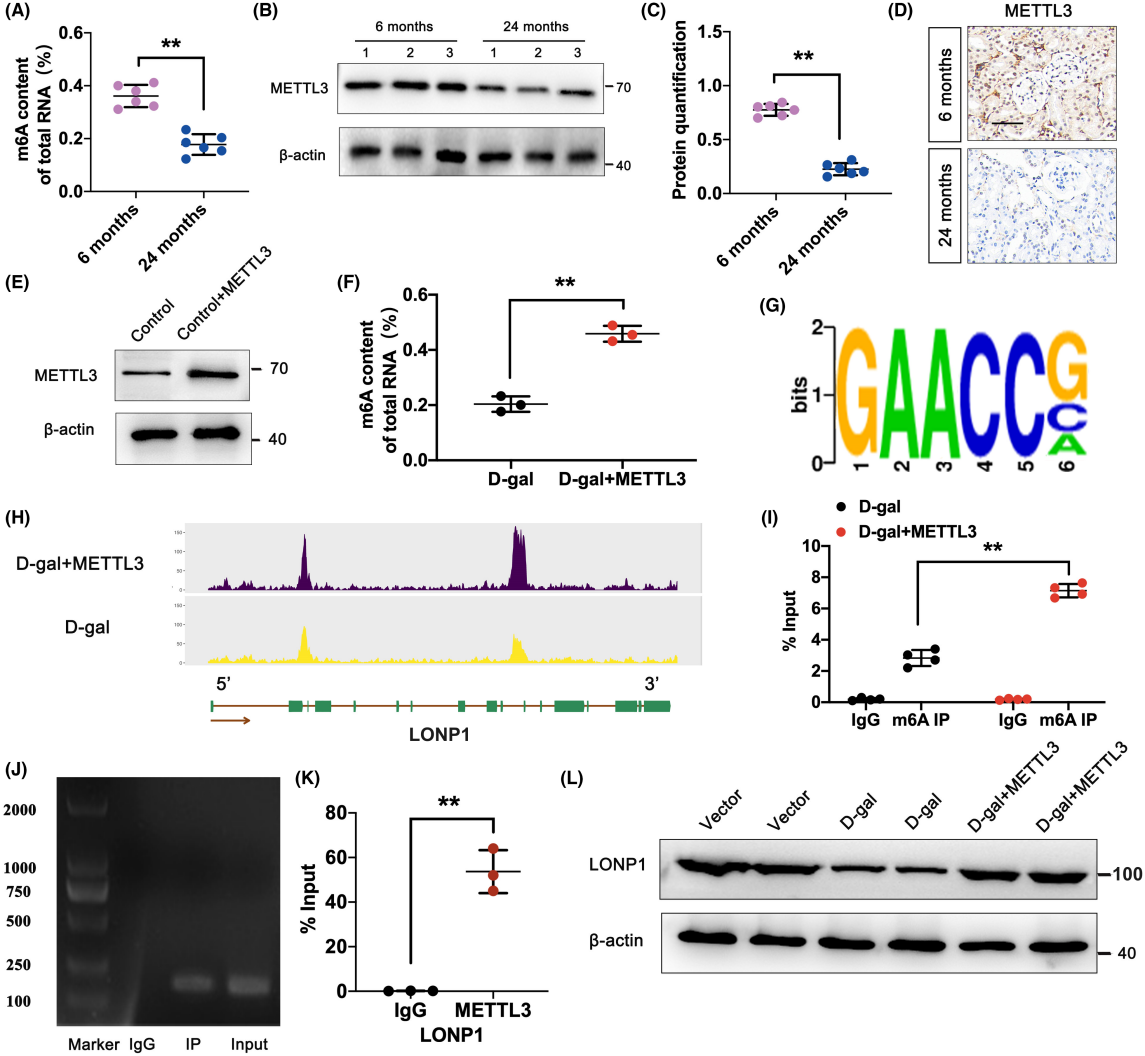
METTL3 regulates the aberrant m6A modification of LONP1. (A) Total m6A RNA levels in the 6‐ and 24‐month‐old groups; (B, C) the protein levels of METTL3 in the 6‐ and 24‐month‐old groups; (D) the expression of METTL3 in the 6‐ and 24‐month‐old groups detected by IHC. The scale bar represents 50 μm. (E) Protein level of METTL3 in HK‐2 cells with or without METTL3 overexpression. (F) Total m6A RNA levels in HK‐2 cells with or without METTL3 overexpression. (G) Motif analysis of METTL3 binding sites using RIP‐seq in both the D‐gal and D‐gal groups transfected with METTL3‐overexpressing cells. (H) Assessment of the abundance of m6A in the LONP1 group. (I) MeRIP‐qPCR was used to determine the enrichment of m6A in LONP1 in the D‐gal group transfected with METTL3‐overexpressing cells. (J, K) RIP‐PCR was used to examine the direct interaction between LONP1 and METTL3. (L) Protein level of METTL3 in the vector, D‐gal and D‐gal+METTL3 groups determined by western blotting. **p < 0.01 versus the 6‐month group (A, C), D‐gal group (F), or IgG group (K), as determined by Student's *t*‐test. METTL3, methyltransferase like 3; LONP1, lon protease 1; IHC, immunohistochemistry; RIP, RNA immunoprecipitation; MeRIP, methylated RNA immunoprecipitation; SD, standard deviation; m6A, N6‐methyladenosine; ANOVA, analysis of variance.

m6A is a common RNA modification in eukaryotes, primarily in messenger RNA (mRNA). Therefore, we tested the m6A modification of LONP1. The levels of m6A methylation were measured in the kidneys of 6‐ and 24‐month‐old mice. As shown in Figure 6A, m6A methylation was significantly downregulated in the aged group (*p* < 0.01). METTL3, an important m6A methylase that has been widely studied in kidney disease, was further examined in the kidney. Compared with that in the 6‐month group, the METTL3 level was significantly lower in the 24‐month group (*p* < 0.01) (Figure 6B–D). We further assessed the functional role of METTL3 in DKD and found that the expression of METTL3 was obviously augmented by transfection with the METTL3 vector (Figure 6E). In addition, the m6A levels were significantly increased by METTL3 overexpression in HK‐2 cells treated with D‐gal (*p* < 0.01) (Figure 6F). Subsequently, we conducted RIP‐seq analysis. The results indicated that METTL3‐mediated mRNA modification in HK‐2 cells predominantly occurred within the ‘RRACH’ nucleotide sequence (R = G or A, H = A, C, or U) (Figure 6G). Visual analysis revealed a substantial upregulation of LONP1 in the 3′UTR when METTL3 was overexpressed in HK‐2 cells compared with the D‐gal group (Figure 6H). Furthermore, MeRIP‐qPCR revealed that the m6A level of LONP1 was significantly greater in cells transfected with the METTL3 overexpression plasmid (Figure 6I), and RIP‐PCR verified that there was a direct correlation between LONP1 and METTL3 (Figure 6J,K). Given the direct interaction between METTL3 and LONP1, the regulatory function of METTL3 was investigated in D‐gal mice by the injection of AAV‐METTL3. The overexpression of METTL3 markedly increased the protein level of LONP1 compared with that in the D‐gal group (Figure 6L). Thus, we demonstrated that dysregulation of the m6A modification of LONP1 was attributed to the abnormal expression of METTL3 during kidney ageing.

### 3.7 METTL3 alleviates mitochondrial dysfunction and renal fibrosis in HK‐2 cells through LONP1 m6A modification

**FIGURE 7:**
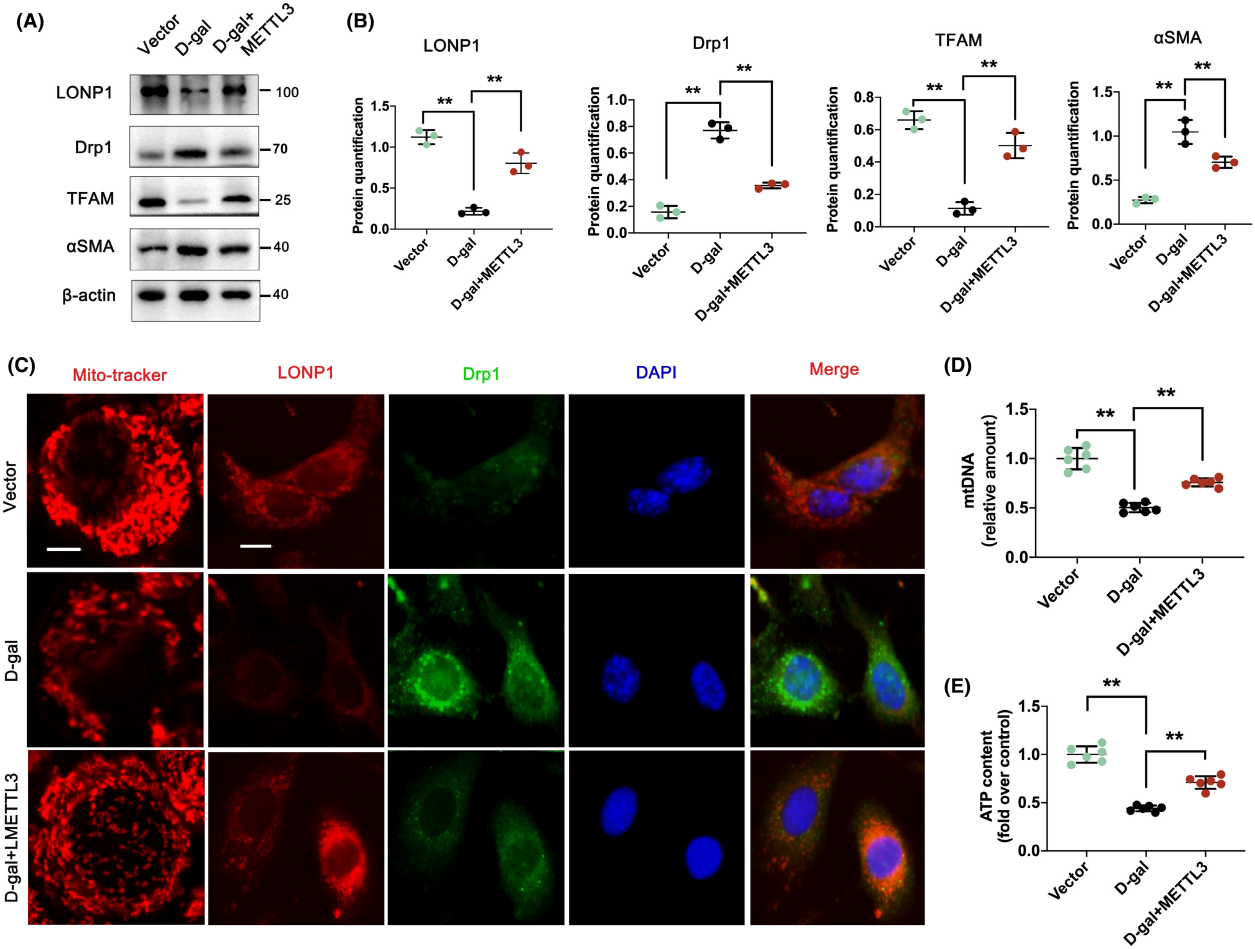
METTL3 alleviates mitochondrial dysfunction and renal fibrosis in HK‐2 cells through LONP1 m6A modification. (A, B) The protein levels of LONP1, Drp1, TFAM and α‐SMA and their semiquantitative analyses in the vector, D‐gal and D‐gal+METTL3 groups; (C) fluorescence micrographs of HK‐2 cells obtained by MitoTracker deep red staining (scale bar = 10 μm) or double immunostaining of LONP1 (red) and Drp1 (green) and counterstaining with DAPI (blue). The scale bar represents 50 μm; (D, E) mtDNA and ATP levels in the three groups of HK‐2 cells. **p < 0.01 versus the D‐gal group by one‐way ANOVA. METTL3, methyltransferase like 3; LONP1, lon protease 1; TFAM, transcription factor A, mitochondrial; Drp1, dynamin related protein 1; α‐SMA, α‐smooth muscle actin; DAPI, 4′,6‐diamidino‐2‐phenylindole; mtDNA, mitochondrial DNA; ANOVA, analysis of variance.

Moreover, we explored whether the functional role of METTL3 in mitochondrial dysfunction and renal fibrosis occurs through the m6A modification of LONP1. As demonstrated in Figure 7A,B, METTL3 overexpression significantly increased the expression levels of LONP1 and TFAM and decreased the expression of Drp1 and αSMA compared to those in the D‐gal group (all *p* < 0.01). Moreover, METTL3 overexpression maintained mitochondrial filamentous morphology, accompanied by increased mtDNA and ATP contents (Figure 7C–E). Double IF staining revealed significantly lower levels of Drp1 and greater levels of TFAM in the METTL3‐overexpressing group than in the D‐gal‐treated group (Figure 7C). These findings revealed that METTL3 might regulate mitochondrial function through LONP1 m6A modification.

### 3.8 LONP1 mRNA stability is enhanced by METTL3 through a m6A‐IGF2BP2‐dependent mechanism

**FIGURE 8:**
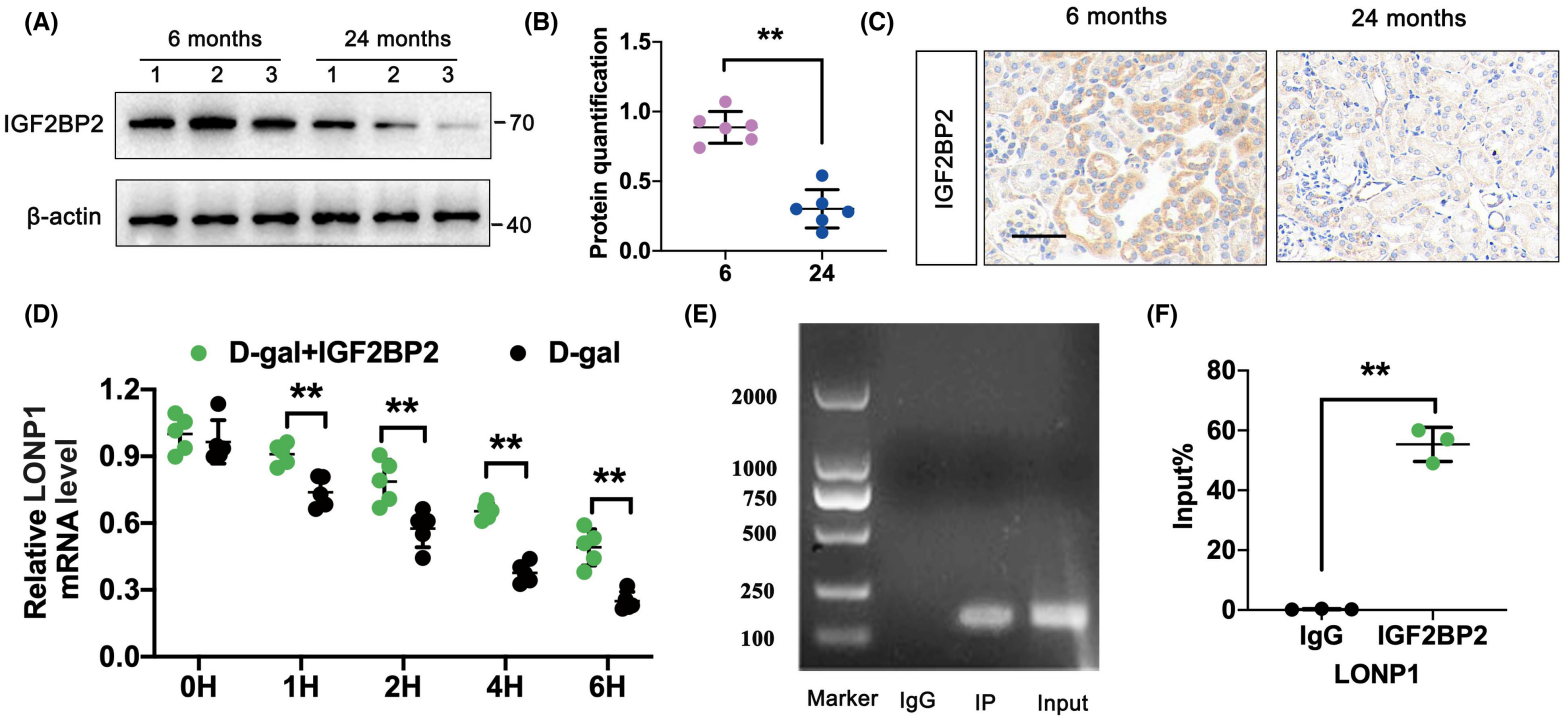
LONP1 mRNA stability is enhanced by METTL3 through a m6A‐IGF2BP2‐dependent mechanism. (A, B) The protein level of IGF2BP2 and its semiquantitative analyses in the 6‐ and 24‐month groups; (C) IHC staining for the expression of IGF2BP2 in the 6‐ and 24‐month‐old groups. (D) The mRNA levels of LONP1 in HK‐2 cells treated with actinomycin determined by RT–qPCR; (E, F) the direct interaction between LONP1 and IGF2BP2. **p < 0.01 versus the 6‐month (B), D‐gal (D), or IgG (F) group according to Student's *t*‐test. LONP1, lon protease 1; METTL3, methyltransferase like 3; IGF2BP2, insulin‐like growth factor 2 mRNA binding protein 2; RIP, RNA immunoprecipitation.

m6A‐regulated RNA is recognized by ‘reader’ proteins, which influence subsequent biological processes. Reports indicate that IGF2BP2, a prevalent m6A reader, targets numerous mRNA transcripts by identifying m6A motifs, thus increasing the stability and translation of mRNAs. Consequently, we found that IGF2BP2 expression was significantly lower in the 24‐month group than in the 6‐month group (Figure 8A,B). IHC staining further confirmed the notably reduced expression of IGF2BP2 in the 24‐month‐old group (Figure 8C). To measure the stability of METTL3‐induced LONP1 under the influence of IGF2BP2, we used actinomycin D for mRNA transcriptional inhibition. Compared with D‐gal, IGF2BP2 overexpression significantly reversed the gradual decrease in LONP1 mRNA in HK‐2 cells (Figure 8D). The RIP‐PCR assay verified the specific interaction between IGF2BP2 and LONP1 mRNA (Figure 8E,F). Our study indicated that IGF2BP2 directly regulates LONP1 mRNA, promoting the stability of LONP1 mRNA in HK‐2 cells through a m6A‐IGF2BP2‐dependent mechanism.

## 4 DISCUSSION

In our study, we showed decreased levels of LONP1 in aged kidneys both in vivo and in vitro. LONP1 deletion significantly accelerated ageing‐related renal fibrosis and mitochondrial dysfunction in HK‐2 cells, while LONP1 overexpression had the opposite effect. Furthermore, we observed abnormal m6A modification of LONP1 by METTL3 and a significant decrease in METTL3 expression, which contributed to renal tubular epithelial cell injury and mitochondrial dysfunction under ageing conditions. Subsequent experiments revealed that decreased levels of IGF2BP2 recognized these m6A sites, impaired LONP1 stability and consequently forced its degradation. Our data suggested that LONP1 might be a potential therapeutic target for the amelioration of kidney ageing.

Ageing significantly contributes to the progression of CKD, as evidenced by the increased incidence of CKD among the elderly population.^4^, ^23^ Additionally, CKD shares characteristics with ageing kidneys, such as glomerular sclerosis, interstitial fibrosis, heightened oxidative stress and sustained inflammation.^24^, ^25^ These findings suggest that CKD can be considered a form of premature ageing.^26^ In the initial phases of CKD, senescent cells tend to accumulate even in patients with mild proteinuria.^27^ The acceleration of senescence and fibrosis mutually reinforce each other, resulting in fragile kidneys developing more advanced pathological features.^28^ Therefore, investigating the underlying pathogenesis of ageing‐related kidney fibrosis is important for identifying crucial therapeutic targets for effectively managing CKD.

Mitochondria serve as the chief energy‐generating organelles in nearly all eukaryotic cells, facilitating oxidative phosphorylation while also playing a crucial role in diverse metabolic processes.^29^, ^30^ Nevertheless, the production of superoxide radicals due to electron transport chain impediments can readily impair the structural function and role of mitochondria, particularly mtDNA, which constitutes the main target of reactive oxygen species (ROS) and is vulnerable to exogenic injury owing to the absence of histone shielding and intrinsic repair mechanisms.^31^, ^32^ Due to the high metabolic demands and energy requirements of the kidneys, they are principally vulnerable to mitochondrial dysfunction. The role of mitochondrial dysfunction in ageing‐related kidney diseases has received increasing attention.^33^, ^34^ Multiple studies have demonstrated alterations in mitochondrial morphology and quantity within PTCs of ageing mice, including reductions in mitochondrial numbers, loss of cristae, enlargement and membrane rupture.^10^, ^35^ In our research, we also observed damaged mitochondrial structures characterized by mitochondria, fragmentation and disorganized cristae. Concurrently, we found that ageing was also associated with the downregulation of ATP content and mtDNA levels. An increasing amount of evidence indicates that mitochondrial dysfunction, characterized by abnormalities in mitophagy and oxidative stress, is involved in the progression of ageing‐related renal fibrosis.^36^, ^37^, ^38^ Mechanistically, the impairment of mitochondria disrupts cellular equilibrium, leading to the overproduction of ROS and the release of proapoptotic factors, ultimately culminating in renal fibrosis. Consistent with these studies,^39^, ^40^, ^41^, ^42^ tubulointerstitial lesions and fibrosis were evident in the aged kidney, concomitant with aberrant mitochondrial quality control.

The Lon protease, the foremost protease in mitochondria, participates in the degradation of aberrant mitochondrial proteins. Knocking out the Lon protease protein reportedly directly results in embryonic death in mice.^43^ Reduced levels of Lon protease lead to the irregular accumulation of mitochondrial proteins, which are responsible for hepatocyte abnormalities,^44^ insulin resistance,^45^ cardiac stress,^46^ bacterial drug resistance^47^ and tumour formation.^48^ Recently, there has been a cumulative indication that LONP1 is responsible for kidney diseases. For example, decreased expression of LONP1 is involved in the pathogenesis of podocytopathy.^20^ Furthermore, the decreased LONP1 in renal tubules may be responsible for the progression of renal fibrosis in CKD patients. LONP1 was reported to play a protective role against the progression of renal fibrosis in two CKD models (unilateral ureteral obstruction and 5/6 nephrectomy) by preserving mitochondrial homeostasis.^16^ Nevertheless, contrary results have also been reported. Liu et al. confirmed that LONP1 levels were increased in the kidney tissues of patients with diabetic kidney disease (DKD) and in an animal model.^49^ Therefore, the functions of LONP1 and the underlying mechanisms need further exploration. In our study, the results revealed that the expression of LONP1 was decreased in kidney biopsy samples from aged people compared to those from younger people, demonstrating a crucial role for LONP1 in renal fibrosis during ageing. Furthermore, for the first time, we revealed that LONP1 impeded the progression of renal fibrosis by alleviating mitochondrial dysfunction in D‐gal‐treated mice and cells after transfection with AAV‐Ksp‐shLONP1 or AAV‐Ksp‐LONP1.

To clarify the mechanism underlying the age‐related reduction in LONP1, we further investigated age‐regulated LONP1 expression at the transcriptional level. m6A modification is the most common modification among eukaryotes.^50^ m6A‐mediated gene expression participates in diverse cellular processes, such as cell apoptosis, organ fibrosis and invasion.^51^ METTL3 is an important methyltransferase responsible for m6A modification. Targeting METTL3 may be a potential way to diminish the development of kidney fibrosis.^52^ In addition, elevated METTL3 levels within podocytes initiate m6A modification of tissue metalloproteinase inhibitor (TIMP)2 mRNA, subsequently facilitating direct interaction between the m6A reader IGF2BP2 and the TIMP2 mRNA m6A site, consequently leading to podocyte dysfunction in DKD.^53^ Considering the abnormal expression of methylated LONP1 in aged kidneys, the downregulation of METTL3 reduced the m6A methylation of LONP1 during kidney ageing. On the basis of the specific interaction between METTL3 and LONP1, the ablation of METTL3 simultaneously reduced the methylation and protein levels of LONP1, accelerating renal fibrosis by maintaining mitochondrial homeostasis. Thus, we concluded that a key mechanism in the control of the LONP1 protein in kidney ageing was METTL3‐induced LONP1 m6A alteration.

The identification of particular m6A sites largely determines the fate of the target transcripts. The mRNA methylation stability and expression of members of the IGF2BP family, which includes IGF2BP1, IGF2BP2 and IGF2BP3, are closely related. These proteins recognize m6A modification sites on mRNA transcripts, which maintains the stability of the transcripts, influences the expression of proteins and serves as functional regulators during times of stress.^54^ In contrast to YTH domain‐containing family protein 2, which promotes mRNA degradation, IGF2BP2 maintains mRNA stability.^55^ When IGF2BP2 detects METTL3‐mediated m6A alterations in MIS12, MIS12 stability is improved, MIS12 expression is upregulated and senescence is reversed in human mesenchymal stem cells.^56^ In our research, a reduction in the expression of IGF2BP2 was detected in the kidney tissue of 24‐month‐old mice. LONP1 mRNA stability is significantly influenced by METTL3 regulation, and IGF2BP2 is directly associated with a specific m6A site, thereby playing a pivotal role in its maintenance under METTL3 control.

While our study utilized an accelerated ageing model to investigate the mechanisms underlying ageing‐related renal fibrosis, several limitations should be acknowledged. The accelerated ageing model employed in this research was designed to replicate specific ageing‐associated pathologies rapidly. Although this approach allows for efficient assessment of interventions within a shorter timeframe, it may not completely reflect the complex biological changes seen in naturally aged organisms. In addition, accelerated models may exhibit exaggerated pathophysiological responses compared to those observed in natural ageing. For instance, tissue damage and metabolic alterations might occur at a pace that does not accurately represent gradual changes in naturally aged individuals. This discrepancy necessitates careful interpretation of our findings when considering their applicability to human populations. Moreover, data derived from accelerated ageing models may differ due to species‐specific responses and inherent biological differences between mice and humans. The multifaceted nature of human ageing—encompassing genetic, environmental and lifestyle factors—means that the effects observed in mouse models may not fully translate to human conditions. Furthermore, natural ageing in mice occurs over an extended period, allowing for more comprehensive investigation of CKD dynamics. To enhance the translatability of our results, further validation in natural ageing models and human samples is essential. Future research will incorporate data from naturally aged mice and human renal samples to enhance understanding of LONP1's role in renal fibrosis.

In summary, our research indicates that LONP1 contributes to ageing‐related renal fibrosis by modulating mitochondrial homeostasis. In addition, LONP1 downregulation is mediated by the m6A methyltransferase METTL3 during ageing. Exploring the regulation of LONP1 as a novel research focus highlights its crucial role in maintaining mitochondrial homeostasis and delaying renal fibrosis.

## AUTHOR CONTRIBUTIONS

**Congxiao Zhang:** Data curation (equal); formal analysis (equal); investigation (equal); resources (equal); writing – original draft (equal). **Siman Shen:** Data curation (equal); formal analysis (equal); investigation (equal); methodology (equal); validation (equal). **Li Xu:** Data curation (equal); software (equal); supervision (equal); validation (equal); visualization (equal); writing – original draft (equal). **Man Li:** Data curation (equal); formal analysis (equal); funding acquisition (equal); software (equal); supervision (equal); visualization (equal). **Binyao Tian:** Data curation (equal); methodology (equal); software (equal); supervision (equal); validation (equal); visualization (equal). **Li Yao:** Conceptualization (equal); project administration (equal); resources (equal); validation (equal); writing – review and editing (equal). **Xinwang Zhu:** Conceptualization (equal); data curation (equal); project administration (equal); supervision (equal); validation (equal); writing – review and editing (equal).

## FUNDING INFORMATION

This study was supported by the Research Project of Health Commission in Shenyang (Grant number 2022024).

## CONFLICT OF INTEREST STATEMENT

The authors declare that they have no competing interests.

## CONSENT FOR PUBLICATION

All authors consent to the publication of this study.

## Supporting information


**Figure S1.** Increased expression of LONP1 transfected with overexpressed LONP1 plasmid in HK‐2 cells. (A, B) Protein level of LONP1 in Vector and LONP1 overexpressed (OE) group by western blot and its semi‐quantitative analysis. **p < 0.01 indicates a significant difference versus Vector group by Student’s *t*‐test.

Table S1.

## Data Availability

The datasets used and/or analysed during the current study are available from the corresponding author upon reasonable request.
