# Supplementary figures and images for: LONP1 alleviates ageing‐related renal fibrosis by maintaining mitochondrial homeostasis
Source: J Cell Mol Med. 2024 Sep 11;28(17):e70090. doi: 10.1111/jcmm.70090 (PMC11390342; doi:10.1111/jcmm.70090)

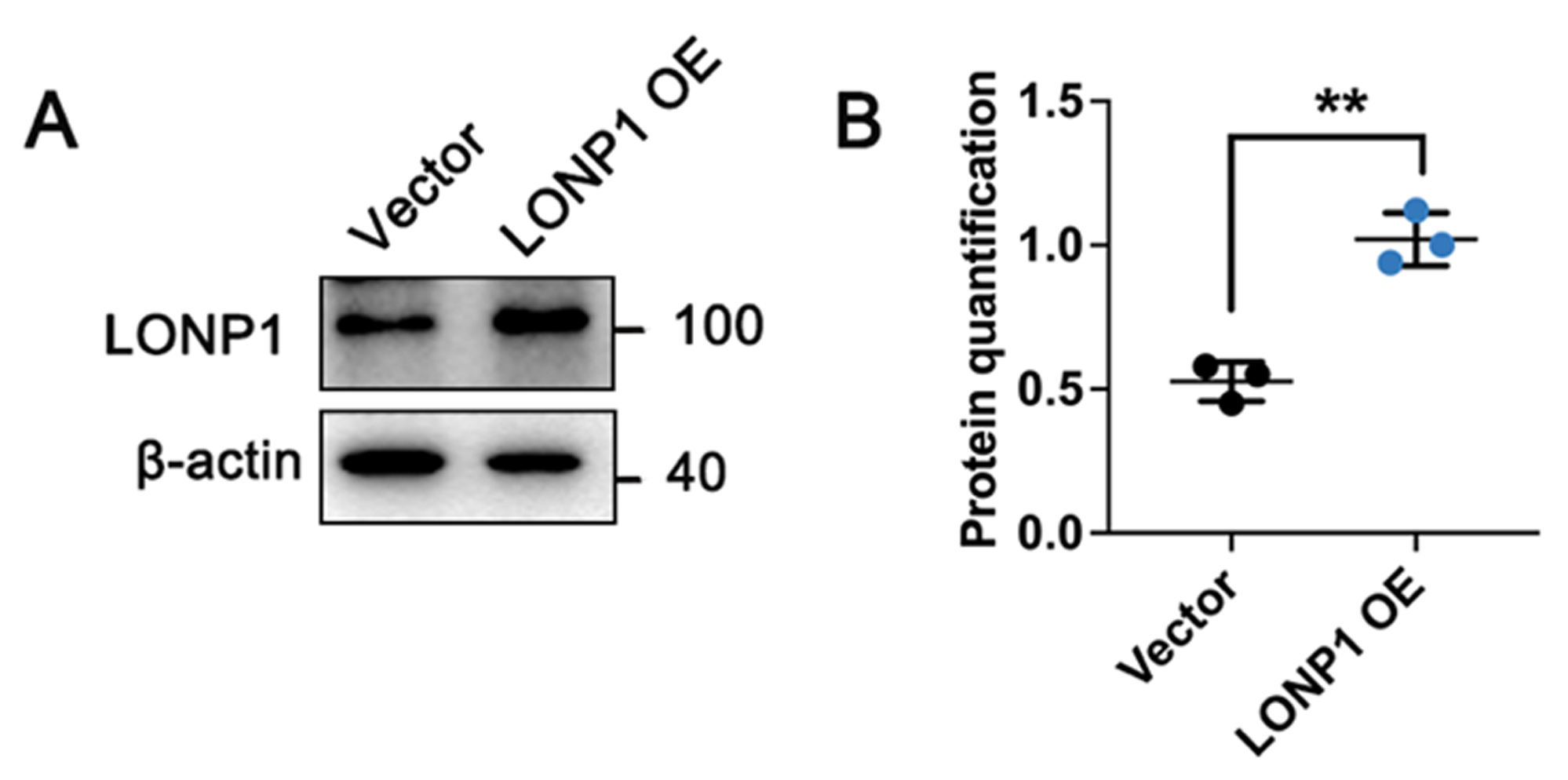

Supplement: Supplementary file 1 — Figure S1. Increased expression of LONP1 transfected with overexpressed LONP1 plasmid in HK‐2 cells. (A, B) Protein level of LONP1 in Vector and LONP1 overexpressed (OE) group by western blot and its semi‐quantitative analysis. **p < 0.01 indicates a significant difference versus Vector group by Student’s t‐test. [file JCMM-28-e70090-s002.tif]
