# Supplementary material for: LONP1 alleviates ageing‐related renal fibrosis by maintaining mitochondrial homeostasis
Source: J Cell Mol Med. 2024 Sep 11;28(17):e70090. doi: 10.1111/jcmm.70090 (PMC11390342; doi:10.1111/jcmm.70090)
Supplement: Supplementary file 2 — Table S1. [file JCMM-28-e70090-s001.docx]

**Table S1. Clinical characteristics of the participants**

| Item | Control (mean ± SD) | Aged (mean ± SD) |
| --- | --- | --- |
| N | 10 | 10 |
| Aged (year) | 40.4±3.9 | 71.4±5.6^***^ |
| Weight (kg) | 72.1±13.9 | 66.3±12.4 |
| Serum creatinine (μmol/L) | 70±14.9 | 79.9±18.2 |
| Urea nitrogen (mmol/L) | 4.43±1.15 | 5.21±1.59 |
| Cystatin C (ml/min) | 0.85±0.14 | 0.93±0.17 |
| Proteinuria | Negative | Negative |
| Gender ratio | 1:1 | 1.5:1 |
